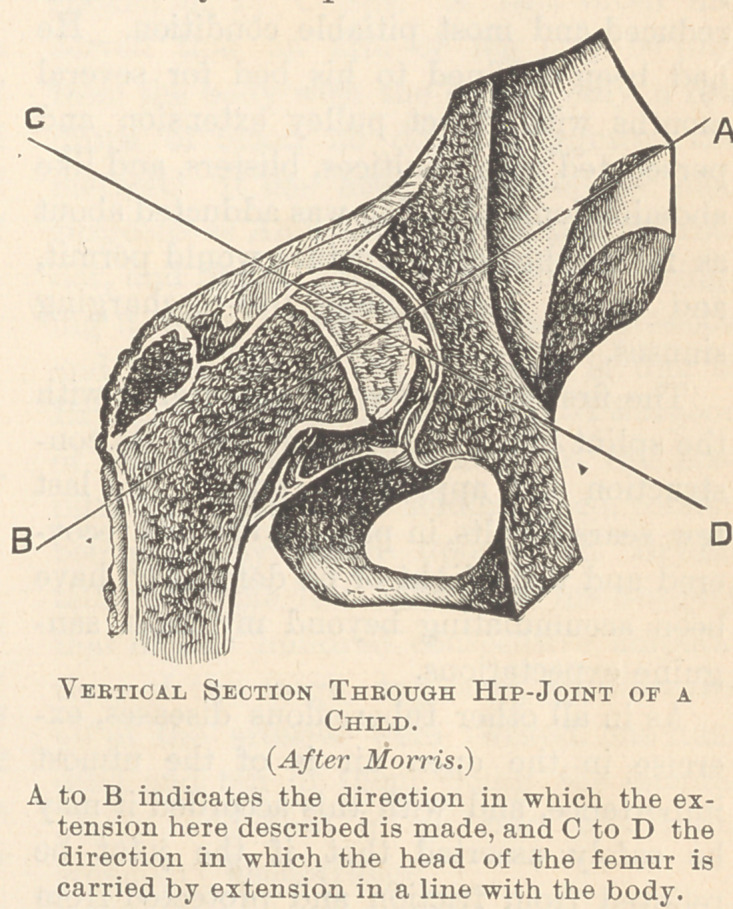# Mechanical Treatment of Hip-joint Disease1Read before the Chicago Medical Society, May 20.

**Published:** 1889-06

**Authors:** Wallace Blanchard

**Affiliations:** 34 Monroe Street


CHICAGO MEDICAL

JOURNAL AND EXAMINER.

VOLUME LVIII. JUNE, 1889. NUMBER 6.

### MECHANICAL TREATMENT OF HIP-JOINT DISEASE.[1]

BY WALLACE BLANCHARD, M.D.

An orthopedic apparatus to be effective should be constructed with the anatomical relations and pathological conditions to be relieved constantly in view. Both these points wonld seem to have been nearly if not quite ignored in the construction of the many forms of extension splints used in hip-disease.

The Sayre, Taylor, Hutchison, Andrews and Bauer splints and their many modifications are all directed to the one object of extending the leg and thigh in a line with the body. This extension added to the power of the large mass of muscles having the pelvis for their base and centering to insertion in the femur, and with the line of their force almost directly inward, must of necessity impinge the head of the femur with augmented force into the lower half of the acetabulum; this really amounts to a partial fixation which is only increased at expense of added pressure in the lower half of the socket, while motion is only slightly impeded. The method of Dr. Hutchison, of Brooklyn, which consists of a high shoe and crutches and allowing the weight of the leg to make its own extension, and Dr. Judson's ischiatic crutch which involves the same principle, are only less mischievous treatments; for the direction of impingement must still be into the acetabulum.

If friction be rightly defined as a combination of motion and pressure, then it is practically undiminished by any of these so-called extension-splints which we too often see patients wearing (and occasionally only under the direction of a mechanic), from the time the inflammatory stage is first discovered, along through the period of suppuration and into the stage of real shortening, until the grinding movement in the socket has so far destroyed the articulating surfaces that the often worse than useless instrument is laid aside and the patient of necessity comes to the last resort, the excision of the head of the femur. And so it happens that the English surgeon, with a sneer at "The American idea of extension," prefers to bind his patient to an inflexible splint extending from shoulder to heel on the affected side, which secures nearly complete immobility. Mr. Thomas of Liverpool introduced some years ago one of the best of this kind, and of late, in this country as well as in Europe, plaster-of-paris and other materials, as also a wire cuirass and a portable bed, have been made to serve the same purpose, but this treatment at best relieves only the other factor of friction—that is, motion.

[1] Read before the Chicago Medical Society, May 20.

It is beyond question that a really efficient apparatus for hip-disease must meet three requirements.

First. It must afford traction outward as well as downward so that the mean force exerted shall be in an axis with the neck of the femur and thus relieve from pressure all the articulating surfaces of the joint.

Second. It must afford immobility.

Third. Allow of unlimited out-of-door exercise, as far as pain or danger to the diseased joint is concerned.

It is submitted that the apparatus here described meets these requirements. It is made mainly of strip iron known as "Binding," and is bent to the form of the body by means of an ordinary monkey-wrench. The main strip should extend from the lower angle of the scapula down over the back, thigh and leg on the affected side to within two inches of the heel. To this are riveted three bands, one to fit loosely around the body just below the armpits, a second at the knee, and the third at the ankle. A spring reaches from the crest of the ilium down over the outside of the thigh to the knee, to which is attached a wide soft padded band passing around inside the thigh, by means of which all the lateral traction which the patient will tolerate is exerted. Extension in a line with the body is had from adhesive plaster applied to the leg and counteracted by a leather belt around the waist to which the loose iron body-band is looped.

The power of both extensions being equal, the mean extension will be seen to be in an axis with the neck of the femur.

The main strip down the back, thigh and leg secures nearly complete immobility, and I have come to look at this as almost as necessary as in fractures. The surface of the splint next to the body is padded and the whole instrument is covered with sheepskin. A patten or cork sole is placed under the foot of the unaffected side, of such thickness as shall prevent the other foot from touching the floor. The patient is given a pair of crutches and told to take all the exercise in the open air that his general condition will permit. The splint should be moulded perfectly to the form and be painless to wear, or it has not been properly applied.

The joint is now protected from jar or concussion and both factors of friction are largely overcome. The adduction of the leg with the accompanying lordosis, which is nearly invariable as the disease progresses, and which is due to the unrelenting tonic contraction of the adductor muscles, is in most cases nearly or quite overcome on the application of the lateral tension band. As the adductors relax and the leg falls into the natural line with the body the upright section of the splint should be straightened so far as to conform to the improved position.

Dr. Sayre asks, speaking of the painful stage, "Why does not the joint accommodate itself to the increased effusion within it?" and answers; "Because there is a constant struggle going on between the over-distended capsule and the adductor muscles." Then what could be more clearly indicated than that the inflamed and ulcerated bone structure should be aided in its struggle for life against the strong and healthy but nervously contracting adductors as is done by the combined lateral and direct extension just described? There are two recognitions of the necessity of a lateral extension in the adduction screw, which is ineffective and never received much favor, and in the adduction crutch which transfers the pressure to the opposite groin and has the disadvantage of hampering the unaffected side.

It is quite unnecessary to try to explain the painful contraction of the adductors by assuming that the obturator nerve suffers from some special irritation as is held by most orthopedic surgeons, when we reflect that the adductors arise from the pubic angle and vicinity to be inserted, taking the three as one, in the shaft of the femur for its whole length, and that they are given the long end of the lever, as it were, against all the opposing spasm-afflicted muscles, and must of necessity tire and relax all resisting muscular forces and exert a crushing influence in the joint.

Views as to the pathology of the disease have undergone radical changes in the past few years, and with a clear idea of the pathological conditions to be met, come indications for improved and more successful treatment.

Briefly stated, a central ostitis is now recognized as the usual lesion. Tubercle is deposited in the cancellated structure of the bone which softens and ulcerates into the cavity of the joint. It appears more than probable that a traumatism primarily often becomes, as the bone softens, a seat for the deposit of tubercle. Indeed, it is certain that there exists an intimate relation between traumatism and tuberculosis independent of the fact that parents and friends are always ready with sufficient evidence to prove the traumatic theory of inception.

Statistics and general experience seem to show an average of about twenty-four per cent. more males than females afflicted with the disease, though my experience would indicate that the males were fully forty per cent. in excess. Mr. Barwell was the first to call attention some eight years ago to the large proportion of hip-disease with phimosis. The clinical reports of Charing Cross Hospital seemed to show that ninety-four per cent. of the males

Vertical Section Through Hip-Joint of a Child.

(*After Morris.*)

A to B indicates the direction in which the extension here described is made, and C to D the direction in which the head of the femur is carried by extension in a line with the body.

admitted with hip-disease were phimosed to a greater or less degree. Though subsequent experience has shown this percentage to be much too large, still frequently an operation for phimosis is indicated.

Perhaps it was excusable to look with a feeling akin to pity on the professional friend who had diagnosed and treated a case of lumbar rheumatism or tabes dorsalis that later came under our observation as a marked case of hip-disease, until Dr. Gibney showed the not infrequent connection between hip-disease and rheumatism, and

M. Charcot demonstrated its occasional neural origin.

About twelve years ago I first applied the apparatus I have described, in a somewhat crude shape, but with a moderately clear idea of what I wished to accomplish, to two boys, each being ten years of age. One was in Mercy Hospital and the other was the son of Mr. H.——, residing on Locust street. The first was soon lost sight of and the second made a good recovery in one year, with hardly noticeable deformity, though when the appliance was first adjusted the little fellow was in a badly reduced and most pitiable condition. He had been confined to his bed for several months with direct pulley extension and persecuted with poultices, blisters, and like abominations. The leg was adducted about as far as the bone structure would permit, and there were three pus-discharging sinuses.

The first year or two of experience with the splint taught improvements in its construction and application, and in the last few years results, in patients entirely recovered and with slight or no deformity, have been accumulating beyond my most sanguine expectations.

As in all other tuberculous diseases, exercise in the open air is of the utmost importance, and with this accorded it may be safely asserted that if the joint be relieved from friction and protected from injury and a generous diet insisted upon, with such medication as each particular case may require, it will be very seldom that a patient taken for treatment in the first stage will reach the second, and the surgeon will frequently be surprised at the rapid recovery of cases taken after they have advanced well into the suppurative stage.

Occasionally a case occurs that is painfully violent in its course and that seems to have a tendency toward a rapid and complete destruction of the joint, but the conclusion must not be drawn that it will necessarily prove less amenable to the treatment herein advocated, for such cases have frequently made a complete recovery in a comparatively short time and with but slight defect, and often none to indicate that the disease was ever present. Neither does it necessarily follow that there is any such special or general tendency to tubercular deposit as will influence a return to a diseased condition either in the joint or other parts of the body.

Confining the patient to the bed with a weight and pulley extension as is advised in many of the text books seems like a relic of barbarism and is sufficient in itself to ruin the health of an otherwise well person. It is seldom necessary even in the painful stage and is practiced only too frequently. There are several points to be noted in the treatment of hip-disease that are so clear that they seem like aphorisms, and I contend that they can only be neglected at the expense of pain, deformity, and danger to the life of the patient.

First. It is friction that destroys the delicate new tissues of repair more rapidly than nature can provide them.

Second. As friction is relieved, so proportionately are the chances of ankylosis reduced.

Third. Every ounce of extension rightly directed relieves an ounce of pressure, though with all the extension which the patient will tolerate there may be no appreciable separation of the head from the acetabulum.

Fourth. Extension prevents jar and concussion in the socket.

Fifth. Extension relaxes the muscles by overcoming reflex spasm.

Sixth. Extension to overcome adduction to the best advantage and at the same time relieve pressure in the joint must be exerted in an axis with the neck of the femur.

Seventh. With proper attention in a given case, improvement will generally be in proportion as movement and pressure, the two factors of friction, are relieved.

Since writing the above an article appeared in *The New York Medical Record* of May 4, from Dr. Phelps of New York, recommending an "Extension corresponding to an axis with the neck" and favoring for that purpose an outside bar-attachment to the old perineal crutch, which is intended to relieve pressure, but not movement. Almost at the same time an English journal came to hand with the declaration that "Movement is a greater evil than pressure."

If this paper has a mission, it is to show that the gentlemen on both sides of the Atlantic are right to a degree, and that when they combine their methods of treatment and thus relieve both factors of friction they will have found the truly rational and really successful treatment for hip-disease.

34 Monroe Street.

## Figures and Tables

**Figure f1:**
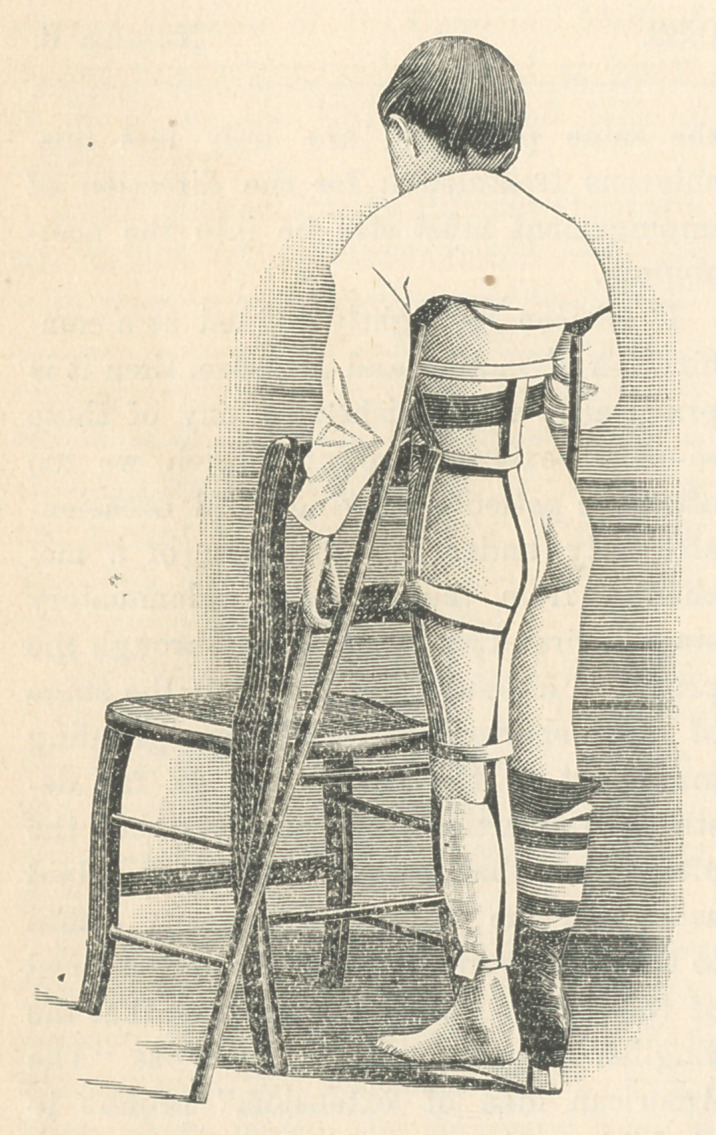


**Figure f2:**